# Hope, Loneliness and Sense of Coherence among Bereaved Parents

**DOI:** 10.3390/ijerph17082797

**Published:** 2020-04-18

**Authors:** Michal Einav, Malka Margalit

**Affiliations:** 1 Behavioral Sciences Department, Peres Academic Center, Rechovot 7610202, Israel; einav.michal@gmail.com; 2 Behavioral Sciences Department, The Academic College of Tel—Aviv-Yaffa, Tel-Aviv 6818218, Israel; 3 School of Education, Tel Aviv University, Tel-Aviv 6997505, Israel

**Keywords:** bereavement, loneliness, hope, sense of coherence, moderated mediation

## Abstract

Coping with the loss of a child is a challenging and difficult experience that disrupts the lives of the surviving parents and the fabric of the family. Our goal is to identify the factors that help bereaved parents cope with this loss and introduce hope and future perspectives into their lives. Our sample consisted of 81 parents (30 fathers and 51 mothers), who completed questionnaires on the following topics: family climate, loneliness, sense of coherence and hope. In addition, interviews were conducted with six parents to further clarify the quantitative results. A moderated mediation model revealed that increased levels of loneliness among the parents predicted lower levels of hope. However, their emotional resources in terms of their sense of coherence mediated this relationship. In addition, the number of years since the loss moderated the negative relationship between loneliness and the parents’ emotional resources. It can be concluded that the negative impact of loneliness on parents’ sense of coherence declined over time. The interviews conducted extend the understanding of these results, as parents described their ability to continue with their lives and identified their goals in terms of the hope theory, alongside their ongoing pain. Finally, the therapeutic implications of the results we obtained are discussed.

## 1. Introduction

### 1.1. Bereavement

Coping with bereavement is regarded as a particularly challenging and difficult experience. The death of a child breaks the natural cycle of life and undermines the perception of life as we know it. The psychological toll of bereavement is grave, both during the immediate response to loss and through the long-term impact on everyday life. People experience grief as a major disruption in their life. They report severe emotional distress, including intense feelings of shock, pain, anger, and guilt. They also find it more difficult to engage in everyday life, reflecting the impact on personal history, personality traits, and environmental conditions [1,2,3]. 

A comprehensive study that explored the loss of partners and parents [4] emphasized that the study of loss must move from examining specific, isolated factors to considering a systemic, dynamic web of intertwined variables and processes that influence each other mutually and dynamically. Consequently, these variables should not be treated as the sum of different, isolated factors, but rather as a dynamic array of mechanisms that activate distress and hinder the resumption of everyday life [5].

Additional studies emphasized the role of avoidance, loneliness and depression as characteristics related to ongoing pain [5]. Although responses to bereavement reflect early personality traits, these characteristics are magnified following the event, impairing the individuals’ coping abilities, adaptation, and quality of life [6]. Research that focused on the avoidance reactions of bereaved parents has produced contradictory results. In several studies, parents reported a decline in their levels of activity, while, in other studies, overactivity [5]. The need to preserve the emotional link to the deceased family member, while struggling to continue with ordinary daily life and developing future perspectives and expectations creates an emotional tension and a sense of distress [6].

Bereavement has been conceptualized as an interruption in the course of life. The identification and development of new resources for creating goals and re-establishing meaning in life are significant in overcoming the avoidance due to bereavement [7]. The hope theory proposes ways to identify approaches for introducing and integrating future perspectives with current behavior.

### 1.2. Hope

The importance of hope for the human experience is clearly evident in art and has been the focus of philosophical and psychological attention. However, in recent years, hope has received research attention as well [8]. The concept of hope reflects the significance of people’s future perspectives for their present circumstances. Even at times of challenging and difficult experiences, the thought of an attainable future can help people adapt, improve their quality of life and reduce the paralyzing impact of emotional distress and traumatic experiences [9].

Hope has been defined hope as “the perceived capability to derive pathways to desired goals and motivate oneself via agency thinking to use those pathways” [10] (p. 249). The hope construct includes two goal-focused factors. Agency thinking focuses on motivations and people’s beliefs in their ability to identify and achieve goals regarded as significant. Pathway thinking focuses on planning the pathways and strategies for achieving those goals. Some pathways involve the direct management of goal requirements, while others identify potential barriers and difficulties, and plan ways of overcoming or bypassing them. Therefore, hope includes the ability to identify meaningful goals and anticipate their future realization. This ability relies on a sense of personal competence, the identification of goals, and the ability to trust one’s coping capabilities, either independently or with external support [8].

Even though, for some people, hope is a natural, intuitive form of thinking that occurs without any external help, others require support, guidance, and certain environmental conditions in order to identify the existence of hope, its potential contribution to their quality of life, and their ability to utilize it when coping with challenging situations. In other words, some people require assistance in order to develop hopeful thinking. This learning process is particularly vital given the positive role that hope plays in various areas such as education, health-promoting habits, and physical and mental health [8,11]. 

There are only a few studies that have examined the links between bereavement and hope. The existing research reports that high levels of hope predict a positive adaptation to life after bereavement [12,13,14]. However, it appears that even though hope is related to wellbeing, the research has not clearly defined the nature of this link. Given that distressing situations such as bereavement reduce people’s expectations about the future, hope, including the ability to outline probable paths to the future, predict possible obstacles, and identify ways of overcoming them, might be a mediating factor between the risk factors of bereavement, adaptation and wellbeing. Therefore, there is an apparent need for in-depth studies that identify the possible mediators between bereavement and hope and examine the personal and interpersonal factors that affect the creation of the latter. Accordingly, studies dealing with personal resources (sense of coherence), interpersonal resources (loneliness) and systematic resources (family climate-cohesion and adaptability) will be reviewed.

### 1.3. Sense of Coherence

People’s sense of coherence reflects the general, global orientation of people’s confidence in the extent to which they have control over the events in their lives, how they understand their world, and the meaning they find in their actions [15]. Sense of coherence is a central concept in the Salutogenic model for promoting health [16]. According to this dynamic model, throughout people’s lives, they move continuously and dynamically along a spectrum of health and illness. Consequently, the model rejects the polarization inherent in the perception of the dichotomous model of health versus illness. Therefore, sense of coherence does not represent a particular style of coping, but refers to a broad repertoire of coping strategies and the flexibility and ability to choose the appropriate one in a given situation.

Sense of coherence is composed of three interrelated factors: (1) comprehensibility—the extent to which individuals consider their environment organized, consistent, structured, and clear; (2) manageability—the extent to which individuals believe that they have access to the sufficient resources (personal or obtained from others) needed for coping with the demands reality imposes; and (3) meaningfulness—the extent to which individuals feel engaged and motivated to cope with the challenges their environment presents [16].

Studies indicate that people with a strong sense of coherence are less vulnerable to stressful situations, as they possess a wide and varied repertoire of coping resources and strategies and are sufficiently flexible and capable of choosing the most appropriate strategy for a given situation. In contrast, people with a weak sense of coherence may exhibit more symptoms associated with stress, experience social difficulties, employ less effective coping mechanisms, and experience loneliness and social isolation [17,18].

### 1.4. Loneliness

Loneliness has been defined as an unpleasant emotional experience occurring when people identify a gap between their available social network and the one they desire [19]. Loneliness expresses dissatisfaction with the quantity and/or quality of interpersonal relationships [20,21]. Adverse emotional, cognitive, and behavioral expressions accompany the experience of loneliness [22] and have a harmful effect on people’s quality of life. Thus, this constitutes a risk factor for adaptation as well as for physical and mental health [23].

Longitudinal studies have identified loneliness as a risk factor for mental health challenges such as depression and anxiety [24]. The research has also established that loneliness tends to persist, so that lonely people tend to remain lonely [21]. Nevertheless, some studies have reported ways of reducing loneliness by changing participants’ perceptions of their sense of control over events and aspects of their life regarding their health, emotions, pleasures, and functionality [25]. In addition, a change in environmental conditions, such as place of residence or social inclusion, may also be catalysts for changes in levels of loneliness [26,27].

Generally, people who are very lonely have reported finding coping very difficult [28] and people who have experienced bereavement at different ages reported intensified feelings of loneliness [6]. Furthermore, research has established that variables such as personality resilience, the nature of the relationship with the deceased, and the context in which they passed away mediate the link between bereavement and loneliness [29]. Therefore, several studies have highlighted the need to consider the multidimensionality of loneliness, including the reciprocal associations among personal, interpersonal, and familial characteristics that reflect the complex, dynamic nature of the phenomenon [30].

### 1.5. Family Climate

The family is regarded as a structured system with rules and norms. All of its members are part of and contribute to the processes that regulates the behavior of its members. This developing, dynamic system consists of interrelated sub-systems as well as extra-familial systems [31,32]. According to this definition of the family, its members influence and are influenced by one another.

Research has identified stages of dynamic development in the family, expressed through the levels of closeness between its members and levels of openness to external influences. Clear boundaries are considered facilitators of family functioning, as they allow the members of the sub-system to fulfill their functions without unexpected disruptions from others [33]. Two major factors affect relationships between these sub-systems and between the members within them: (1) cohesion, meaning the levels of closeness between the family members and the personal autonomy of an individual within her/his family; and (2) adaptability, meaning the family system’s ability to change and be flexible in their reaction to environmental and personal forces. Therefore, a family’s effective functioning depends on both factors: the level of cohesion within it and its ability to adapt to various changing situations while supporting its members’ emotional needs [33]. Studies dealing with family dynamics in situations of bereavement have reported that a family climate that is more cohesive and promotes open communication as well as close, supportive relationships consistently moderates the grief process and helps its members adapt to the new situation [34].

### 1.6. The Purpose of the Study

The current literature review identified the role of hope (within the hope theory) as a multidimensional, dynamic conceptualization of factors that promotes the functional management of challenging situations. Our goal is to identify the factors that can increase hope among bereaved parents. Therefore, in this study, we examine people’s personal resources (their sense of coherence), loneliness as a measure of their interpersonal relationships, and the participants’ family climate within the area of systemic resources. We explore the links between these variables and identify their relative contribution in predicting the hope of bereaved parents. A small group of parents were interviewed to gain an understanding of the subjective perceptions of the studied variables (loneliness and hope).

## 2. Method

### 2.1. Participants

Eighty-one families of bereaved Israeli parents (30 fathers and 51 mothers) participated in the study. They live in the central–south part of Israel, their son or daughter passed away during their army service, and they receive support from the same social services in the region. However, they were not related to one another. From every family, only one parent (a mother or a father) participated in the study. Families whose loss was shorter than a single year were excluded. Participation was voluntary and the participants consisted of families who lost one child, and whose loss took place at least 12 months ago. Families were contacted to get their consent, and they provided their agreement to participate in the study. The average age of the parents was 58.88 (S.D = 7.89) and the average range of years after the loss was 7.79 (S.D = 3.46). A total of 53 participants (65.4%) were married and their mean number of children was 4.04 (S.D = 1.38). A total of 25 parents (30.9%) reported high school level education, and 50 subjects (61.7%) were employed. Table 1 lists the distribution of the participants according to their personal characteristics.

### 2.2. Measures

#### 2.2.1. Hope

A Hebrew adaptation [35] of the Hope Scale [10] was used to assess hope as defined in the hope theory [10]. The scale consists of six items to which participants responded on a six-point Likert-type scale, ranging from one (never) to six (all of the time) (e.g., “I can think of many ways to get things in life”). Higher scores reflect higher levels of hope. In the current study, a Cronbach’s alpha of 0.85 was obtained.

#### 2.2.2. Sense of Coherence (SOC)

The short version of the self-report scale [16] was used to rate parents’ sense of confidence in themselves and in their world, their sense of how they understood their environment, their sense that they could manage their environment and control it, and their sense of meaningfulness. The scale consisted of 13 items rated on a seven-point Likert-type scale, ranging from one to seven. For example, statements such as “Doing the things you do every day is” were rated using descriptors such as “a source of pain and boredom” (one) to “a source of deep pleasure and satisfaction” (one). Higher scores reflected higher levels of coherence. Cronbach’s alpha for internal consistency in the current study was 0.82.

#### 2.2.3. Loneliness

The Hebrew adaptation [36] of the loneliness scale [37] was used. It consists of 11 statements describing social and emotional loneliness. Items include: “I miss having a really close friend,” and “I often feel rejected”. The measure uses a one (not appropriate) to five (very much) scale. The Cronbach’s alpha in the current study was *α* = 0.85.

#### 2.2.4. Family Climate

The Hebrew adaptation [38] of the Family Climate Scale (FACES III; [31]), assessed the degrees of cohesion and adaptability within the family. The questionnaire asks respondents to indicate on a Likert scale ranging from one (almost never) to five (almost always) the number that most appropriately describes their family with regard to a variety of statements. The cohesion subscale consists of 10 items (e.g., “Family members feel closer to other family members than to people outside the family”) and measures emotional bonding, family boundaries, and time spent together. The adaptability subscale consists of 10 items (e.g., “We shift household responsibilities from person to person”) and measures the extent to which the family system is flexible and able to change. Higher scores reflect higher levels of cohesion and adaptability. Research supports the reliability and validity of the FACES III scale [31]. In the current study, a Cronbach’s alpha of 0.74 was obtained for the cohesion subscale and 0.72 for the adaptability subscale.

#### 2.2.5. Interviews

Social workers conducted six semi-structured interviews with parents who participated in the study. They asked the parents to share their personal story of the loss they experienced and indicate the aspects that were most important when coping with the loss. The parents were encouraged to describe the factors that helped them during their most difficult periods. The social workers ended the interviews with the question “Based on your experience, what can help other parents in a similar situation?”. We were able to interview only six parents since the sample in this study consisted of a vulnerable group, and the studied topic is painful. Each one of the social workers was able to interview a single parent. However, in line with several methodological approaches [39,40], an inconsistency was proposed regarding the sample size in the qualitative part of this mixed design study.

#### 2.2.6. Procedure

First, the researchers met with the group of social workers, who work regularly with these bereaved families, to discuss the research model and procedures according to ethical regulations in order to protect the anonymity and feelings of the vulnerable families. Families that lost their son or daughter under 12 months ago were excluded. Afterwards, the research proposal and the questionnaires were submitted to the Ethics Committee of the Peres Academic Center. Following the committee’s confirmation that the research proposal met all the ethical principles in behavioral research, a letter was sent to parents asking for their participation in the study. Only one parent from each family (according to their choice) participated in the study. Following the parents’ consent to participate in the study, the families received the questionnaires, and returned them in sealed envelopes without any means of identification.

After the questionnaires were collected, each one of the social workers selected a family that they considered a typical family, and interviewed a single parent from that family at their home. Only parents who expressed their agreement to be interviewed participated in the interviews, with the stipulation that the interviews would not be connected to the anonymous questionnaires’ results.

## 3. Data Analysis

### 3.1. Quantitative Analysis

We conducted the analyses using IBM SPSS 25 (IBM, Armonk, NY, USA) for Windows. In the first stage, we conducted preliminary analyses, including calculating Pearson’s correlations to examine the associations among the research measures. To further explore the relations among the predictor variables, and to identify a mediating and moderating path, we used the bootstrapping approach [41]. Specifically, we used Model 7 in the PROCESS macro, which is a moderated mediation model. In accordance with the recommendations, all regression/path coefficients were in unstandardized form and the variables were mean-centered prior to analysis.

Before turning to the results, it is important to add a word of caution about the language used in reporting our findings. The results are conveyed in terms of associations rather than effects, as causal effects cannot be established without controlled experimental data. However, one of the important features of moderated mediation analysis is that it allows us to draw distinctions among direct, indirect, and total effects. Thus, we have chosen to use the word “effect” when discussing the results, but its use does not imply that causality has been established.

### 3.2. Qualitative Analysis

Each interview was transcribed verbatim. Two social workers read each one of the interviews separately and listed the themes and motives that were raised in the interview, and afterwards compared the themes. We used a deductive and inductive thematic analysis approach [42,43]. Thematic analysis is a method for identifying, analyzing, and interpreting patterns of meaning called themes within qualitative data. Although we expected preconceived themes from the interviews, while listening to the audiotaped interviews, we identified initial discursive themes and codes. We grouped the themes together and then checked for emerging patterns, variability and consistency, and the functions and effects of specific discourses, then compared them to the quantitative results. The interpretation of these themes was conducted by reading and re-reading the interviews, and referring to the results of the quantitative analyses and the relevant literature. Once coding was complete, the data were examined for differences and commonalities both within and across code categories and in comparison with the quantitative analysis.

## 4. Results

### 4.1. Preliminary Analyses

Table 2 lists the Pearson’s correlations among the research measures. The results demonstrated that the relationships between hope, sense of coherence and loneliness were significant. Family cohesion was positively related to family adaptation and negatively related to loneliness and to the number of years since the loss.

### 4.2. Moderated Mediation Model

To explore the relationships between the predictive variables and hope, we tested a moderated mediation model (Model 7) using the PROCESS macro [41]. Indirect effects were tested using 5000 bootstrapped resamples. Loneliness was entered as a predictor, sense of coherence as a mediator, time since the loss as a moderator, and hope as an outcome. To determine whether the prediction of loneliness on sense of coherence was moderated by the time that had passed since the loss of a son or daughter, we followed the recommendations and used a regression-based approach with the bootstrap method [41]. In this approach, non-standardized beta coefficients are calculated in order to reduce Type 1 errors. Initially, the total effect of experiencing loneliness on hope was at a significant level (*C* = −0.34, SE = 0.09, t = −3.72, *p* < 0.01). However, the mediator variable model revealed a significant loneliness and ‘years after the loss’ interaction on sense of coherence (b = 0.26, SE = 0.01, t = 2.68, *p* < 0.01), and a significant main effect of sense of coherence on hope (b = 0.60, SE = 0.12, t = 5.15, *p* < 0.00). As expected, the indirect effect of loneliness on sense of coherence decreased as the time since the loss increased. The significance of this model was verified with bootstrapped standard errors and 95% confidence intervals (CIs).

**Figure 1:**
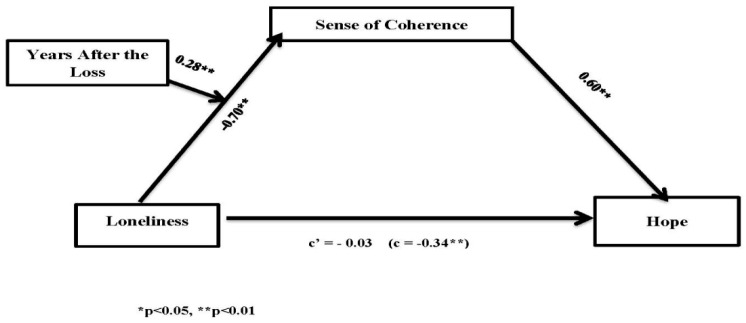
The moderated mediation of the relationship between loneliness and hope.

Figure 1 illustrates the moderation of time since the loss and the mediation of the sense of coherence on the relationship between loneliness and hope. Based on these results, we concluded that personal resources such as sense of coherence, moderated by the time since the loss, fully mediated the negative relationships between loneliness and hope. Overall, the model was significant (F (2, 73) = 23.78, *p* < 0.01).

### 4.3. Thematic Analysis

The thematic analysis focused our attention on the personal meaning of the themes of loneliness, coping, hope and passing time.

### 4.4. Loneliness

The parents described the loss as a physical pain, a feeling that they had lost a part of their body: “I feel that the pain is almost physical. Almost as if I lost a limb”. They related it to a feeling of social seclusion regardless of their social relations: “The feeling of loneliness is very difficult, although I have the family with me and friends, especially in the evenings and during holidays”. They explained how they struggled with the desire to remain alone with their pain, and the knowledge that they should struggle against this, and avoid social isolation: “I don’t let my husband and kids stay in their corners... we have to stay together”. Another mother stated that “Although it is difficult, don’t isolate yourself from the world”. Some parents shared the importance of relationships with family and friends, and wanted “to stay together and to share the sorrow”. Others reported that sometimes they felt that “their friends feel uneasy to be with them” and said, “Some friends start avoiding us since they don’t know how to deal with our pain”. Others said “Don’t let parents stay alone by themselves”. One father emphasized that “Although it is difficult, don’t isolate yourself from the world”.

### 4.5. Coping and Time

The major dilemma that the parents were struggling with was their ability to find the strength to continue their life, to identify meaningful activities and important commitments. They reported that coping with pain was eased by a focused effort on goals that promoted an active lifestyle. The parents shared their different options: “You need to be active, to work, to study”. “I almost graduated law school”. “My goal is to help my children and grandchildren succeed”. Their ambivalent statements about hope demonstrated their inner struggles: “I don’t have any hope…I hope that my children will be successful”.

Some parents discussed their views on the passing of time. A father said: “People don’t understand that the time that passed does not make a change …there is no hope for me”. Another mother said, “I saw a mother who lost her son 45 years ago with the same pain in her eyes like mine —after 4 years”. “From day one I decided to continue; I have hope and tomorrow. However, from day to day it is not easier or more difficult”. The interviewed parents said that “time does not reduce the pain”. Yet, another mother stated that, “You learn how to continue to live with the pain like two parallel lines; you live the loss and at the same time, you continue with your life. But there are days when the lines meet”.

## 5. Discussion

The purpose of this study was to examine the links between bereaved parents’ loneliness and their levels of hope, while addressing the significant challenges they face. To do so, we explored the factors that might predict hope: sense of coherence (as a personal resource), levels of loneliness (as an interpersonal resource), and family climate (as a systematic resource). Identifying these factors is important in line with many studies that have indicated the immediate and long-term consequences of the loss of a son or daughter. The rift created in the family fabric impacts many significant aspects of both the individual’s life and the family’s ability to protect its members.

The results indicated that higher levels of loneliness were associated with lower levels of coherence and family cohesion. These links emphasized the importance of sense of coherence as a personal resilience factor that predicted hope—the ability to continue, identify future goals, and strive to achieve them. The interviews further clarified these results with statements from the bereaved parents recognizing the need to continue life, without ignoring their pain and loneliness. The interviews extended our understanding of the results, by illustrating that the experience of loneliness is associated with different types of coping and adaptation abilities.

An inverse relationship between the time elapsed since the loss and the level of family cohesion reported by the participants was identified. In addition, the moderated mediation model further clarified that, although a high level of loneliness predicted a weaker sense of coherence, as time passed, the negative impact of social isolation decreased. This finding raises concerns about the potential consequences of family loss, which might not diminish or lessen with the passage of time. Nevertheless, the parents developed the ability to continue with their lives, activate their abilities, and find new goals. Perhaps the negative relationship between time and family cohesion reflects the increase in the individuals’ abilities to fulfill their autonomic goals. However, given that this variable also reflects parental age, the decrease in cohesion may also represent a normative phase in the family lifecycle, the “empty nest” phase, where the emphasis is on promoting personal autonomy rather than on family cohesion [44].

The importance of the time elapsed since the loss is also prominent when examining the results of the moderated mediation model. First, as predicted, levels of loneliness predicted levels of hope. Therefore, when bereaved parents feel less lonely, they also feel more hopeful. However, the individual’s sense of coherence mediated this link. Therefore, people’s existing sense of control over their lives, their understanding about what is happening in their world, and the meaning they find in their involvements all reflect a consistent, personal resource that they can rely on and utilize to help them through their bereavement. This mediating effect is of great importance because it offers the bereaved parents a personal resource they can draw upon to promote hopeful thinking.

The model also suggests that the link between loneliness and sense of coherence is moderated by the amount of time that has elapsed since the loss. Therefore, the more recent the loss, the more loneliness impacts the bereaved parents’ ability to maintain their sense of coherence as a resource of resilience in times of stress. In contrast, the longer the time that has elapsed since the loss, the less loneliness undermines their sense of coherence, thus strengthening and consolidating their sense of hope.

### Limitations of the Study

This study has several limitations. The sample is rather small, drawn from only one region in Israel, with a majority of mothers versus fathers. To be able to generalize our results, we need broader samples from different locations and cultures, with an equal number of mothers and fathers. Another limitation comes from the one-time data collection, which provides only a snapshot of the situation in the present moment. Longitudinal research is needed to examine developmental processes and identify possible changes in different periods, such as personal and national memorial days, birthdays, and family celebrations. Finally, this study was based on self-report questionnaires. An additional limitation is the small number of interviews that were performed. Future studies must incorporate more in-depth interviews in order to fully understand how bereaved parents deal with their loss and their unique, individualistic expressions of feelings, expectations, concerns and hopeful thinking over time.

## 6. Conclusions

Our results underscore the significant links between hope, personal coherence, and loneliness among parents in bereaved families. These components constitute a complex mosaic of factors related to vulnerability, risk, and resilience that significantly impact parents’ ability to cope even in the face of the severe adversity that has befallen them. This multifaceted examination is of great importance when we translate the research findings into practical goals and methods of intervention that may eventually become part of the toolbox of those who care for bereaved families. Identifying both the risks and strengths of the parents will help define specific goals that are tailored to the family’s growth. Given our results, we recommend the adoption of therapeutic approaches developed within the hope theory framework in formulating interventions and building “communities of hope” in an effort to reduce the agony of bereaved parents and boost their personal and family empowerment.

## Figures and Tables

**Table 1:** Participants’ distribution according to demographic variables.

Variable	No.	Percentile (%)	
**Gender**			
Fathers	30	37.04	
Mothers	51	62.96	
**Marital status**			
Married	53	65.4	
Couples	3	3.7	
Divorced	9	11.1	
Widowed	6	7.4	
Not reported	10	12.3	
**Education**			
High school	25	30.9	
Diploma	15	18.5	
Academic	27	33.3	
Not reported	14	17.3	
**Employment**			
Employed	50	61.7	
Non-employed	9	11.1	
Pensioners	18	22.2	
Not reported	4	4.9	
	**Mean**	**Deviation Standard**	**Range**
**Age**	58.88	7.89	80–41
Time Since loss	7.79	3.46	16–1
Number of children	4.04	1.38	8–1

**Table 2:** Pearson correlations between the research variables.

Variables	1	2	3	4	5	6
1. Years after the loss	___					
2. Loneliness	0.13	___				
3. Hope	0.08	−0.39 **	___			
4. Sense of Coherence	0.11	−0.62 **	0.61 **	___		
5. Family Cohesion	−0.28 *	−0.33 **	0.12	0.18	___	
6. Family Adaptability	−0.10	−0.16	−0.05	0.02	0.58 **	___

Notes: * *p* < 0.05, ** *p* < 0.01.
